# Purchasing policy, quarantine and acclimation practices of breeding gilts in Belgian pig farms

**DOI:** 10.1186/s40813-021-00205-2

**Published:** 2021-03-08

**Authors:** Elise Bernaerdt, Jeroen Dewulf, Robin Verhulst, Caroline Bonckaert, Dominiek Maes

**Affiliations:** 1 Unit of Porcine Health Management, Department of Reproduction, Obstetrics and Herd Health, Faculty of Veterinary Medicine, Ghent University, Salisburylaan 133, 9820 Merelbeke, Belgium; 2 Veterinary Epidemiology Unit, Department of Reproduction, Obstetrics and Herd Health, Faculty of Veterinary Medicine, Ghent University, Salisburylaan 133, 9820 Merelbeke, Belgium; 3 Animal Health Care Flanders, Hagenbroeksesteenweg 167, 2500 Lier, Belgium

**Keywords:** Purchasing policy, Quarantine, Acclimation practices, Introduction procedures, Vaccination, Breeding gilts, Pigs

## Abstract

**Background:**

The breeding population is very important in pig herds, for productivity, health and profitability. Replacement of breeding animals can be accomplished by own rearing of breeding gilts or by purchasing them. Purchasing breeding gilts is a hazardous event in terms of biosecurity and introduction of pathogens into a farm. However, in literature, little is known about gilt introduction in a herd. The present study investigated the introduction procedures of purchased breeding gilts in Belgian pig herds, and the compliance of these herds to the optimal introduction procedures. A questionnaire consisting of twenty questions related to farm characteristics (*n* = 2), purchasing policy (*n* = 6), quarantine period (*n* = 5), and acclimation practices (*n* = 7) was designed, and 68 farms completed the questionnaire during an on-farm interview.

**Results:**

The median (min. – max.) number of sows on the farms was 300 (85–2500). Fifty-seven per cent of the farms purchased breeding gilts, and there was a lot of variation in the frequency of purchase and the age at which gilts are purchased. On 95 % of those farms, a quarantine unit was used, and on most of these farms the quarantine was located on the farm itself (internal quarantine). The median (min. – max.) duration of the quarantine period was 42 (14–140) days. The most common acclimation practice was vaccination against Porcine parvovirus (96 %) and *Erysipelothrix rhusiopathiae* (94 %), although in some farms exposure of gilts to farm-specific micro-organisms was done by providing faeces from suckling piglets (18 %) and bringing gilts in contact with sows that will be culled (16 %). Only 10 % of the farms complied with the optimal introduction procedures, i.e. purchasing policy, quarantine building and quarantine management.

**Conclusions:**

This study showed that in many farms, practices related to purchasing, quarantine and acclimation could be improved to maintain optimal biosecurity.

## Background

The breeding population is very important in pig herds, for productivity, health and profitability [1]. Replacement of breeding animals can be accomplished by own rearing of breeding gilts or by purchasing them. Purchasing of breeding gilts might lead to faster improvement of genetic potential, but it includes the risk of pathogen introduction in a farm [2]. For 14 bacteria and 10 viruses causing diseases in swine, transmission by direct contact has been described, i.e. transmission by secretions and excretions of live animals or cadavers [3]. For example, the transmission rate of *Actinobacillus pleuropneumoniae* is estimated to be 10 times higher for direct contact in comparison to indirect transmission by people, semen, manure, rodents, aerosol, feed, water or fomites [4]. Purchasing breeding gilts was found to be a risk factor for seroprevalence of *Mycoplasma hyopneumoniae* in slaughter pigs on farrow-to-finish pig herds [5]. Therefore, purchasing breeding gilts is a hazardous event in terms of the introduction of new pathogens into a farm. Several factors should be taken into account such as frequency of purchase, number of animals purchased, number of origin herds, the transport vehicle, and the health status of the origin farms [6].

Placing purchased animals in a quarantine unit aims to (1) reduce the risk of pathogen introduction into the farm and (2) to facilitate the introduction of the animals into the herd by means of acclimation. During the quarantine period, pigs can be observed for the presence of clinical signs and be tested for the presence of pathogens. Acclimation practices such as vaccination against several pathogens and exposure to live animals (e.g. pigs or sows before culling), can protect newly purchased animals against pathogens circulating on the farm [2, 7].

Based upon guidelines described in the literature, optimal introduction procedures in terms of good biosecurity, can be divided into three main categories, namely purchasing policy, quarantine building and quarantine management [8] From a biosecurity viewpoint, purchasing animals constitutes a risk that can only be minimised, but not completely eliminated. If animals need to be purchased, the farmer could pay attention to the following items to minimise the risk of pathogen introduction: the pigs should always originate from the same farm, the health status of the origin farm should be higher than or equal to the farm, and strict hygiene measures should be implemented for the transport vehicle. Once the newly purchased pigs arrive on the farm, there are some requirements for the building and the management of the quarantine unit. A quarantine unit should be present, and ideally it should be completely isolated from the other animals, with a separate entrance and a separate hygiene lock. The all-in/all-out principle should be practiced, so that new gilts can only arrive when the quarantine unit is completely empty. The duration of the quarantine period should be a minimum of 28 days [8].

The present study investigated the introduction procedures of gilts in Belgian pig farms as a first step to optimise the health management of breeding gilts. We focused on purchasing policy, quarantine period, and acclimation practices. The results were compared to the optimal situation, to determine to what extent these practices are in line with the recommendations for optimal introduction procedures [8].

## Materials and Methods

### Design of the questionnaire

The questionnaire was designed based on the principles outlined in the chapter Questionnaire design in Veterinary Epidemiologic Research [9]. The questionnaire was kept short and not too complex, to lower the response burden and thus increase the response rate. To identify confusing or ambiguous questions, the questionnaire was pre-tested and evaluated by veterinarians of the Unit of Porcine Health Management (Faculty of Veterinary Medicine, Ghent University) (*n* = 4), veterinarians from Animal Health Care Flanders (*n* = 3), and pig practitioners (*n* = 1). Based on their feedback, some final changes were made.

The questionnaire was limited to 20 questions, and some questions from the risk-based biosecurity scoring tool Biocheck.UGent™ were included [10]. First, some general information was asked, such as herd size and the batch management system. Further, the questionnaire was divided into three parts namely purchasing policy, quarantine period and acclimation practices of breeding gilts, each consisting of six, five and seven questions, respectively, regarding the past year. Most questions were multiple choice questions, sometimes ‘fill-in-the-blank’ questions were used to request additional information or to collect numerical data. A checklist was used for some questions. In case of breeding gilts were not purchased, subsequent questions related to purchasing policy and quarantine were skipped. The different questions of the questionnaire are shown in Tables 1, 2 and 3, and 4.

**Table 1:** Results of the categorical variables related to purchasing policy of breeding gilts in pig farms

Variable	n	%
Are breeding gilts purchased? (*n* = 68)		
*Yes* *No*	*39* *29*	*57* *43*
Do you always work with the same origin farm or do you cooperate with multiple? (*n* = 39)		
*Always same origin farm* *Multiple origin farms*	*38* *1*	*97* *3*
Do you have information on the health status of the origin farm? (*n* = 39)		
*Higher than or equal to own farm* *Lower than own farm or not known*	*31* *8*	*79* *21*
Are there hygienic requirements for the transport truck of purchased breeding gilts? (*n* = 39)		
*Yes* *No*	*25* *14*	*64* *36*

**Table 2:** Results of the categorical variables related to the quarantine period of breeding gilts in purchasing farms

Variable	n	%
Is a quarantine unit present at the farm? (*n* = 39)		
*Yes* *No*	*37* *2*	*95* *5*
What is the location of the quarantine unit? (*n* = 37)		
*External – followed by internal quarantine* *External – adding gilts immediately to the herd* *Internal – isolated stable* *Internal – separate compartment within a stable* *Internal – together with other pigs on the farm*	*1* *0* *23* *13* *0*	*3* *0* *62* *35* *0*
Is the all-in/all-out principle used in the quarantine unit? (*n* = 37)		
*Yes* *No*	*32* *5*	*86* *14*
Do you have a separate hygiene lock for the quarantine unit? (*n* = 37)		
*Yes* *No*	*20* *17*	*54* *46*

**Table 3:** Results of the categorical variables related to the acclimation practices of breeding gilts in pig farms

Variable	n	%
Against which pathogens do you vaccinate the breeding gilts? ^a,b^ (*n* = 68)		
*PPV* *PRRSV* *E. rhusiopathiae* *SIV* *PCV-2* *M. hyopneumoniae* *A. pleuropneumoniae* *P. multocida and B. bronchiseptica* *G. parasuis* *PRVA* *E. coli* *Clostridium spp.*	*65* *59* *64* *44* *45* *48* *27* *40* *31* *14* *31* *18*	*96* *87* *94* *65* *66* *71* *40* *59* *46* *21* *46* *26*
Which acclimation practices do you use? Contact with… ^a^ (*n* = 68)		
*Sows that will be culled* *Placenta tissue* *Faeces from suckling piglets* *Faeces from weaned piglets* *Faeces from piglets with diarrhoea* *Other* *None*	*11* *6* *12* *2* *1* *21* *29*	*16* *9* *18* *3* *1* *31* *43*
Are breeding gilts monitored for specific pathogens? ^a^ (*n* = 68)		
*Yes, for Brachyspira hyodysenteriae only* *Yes, for other pathogens than B. hyodysenteriae* *Yes, for B. hyodysenteriae and other pathogens* *No*	*2* *7* *2* *57*	*3* *10* *3* *84*
How are breeding gilts housed? (*n* = 68)		
*Individual housing* *Group housing* *Combination of individual and group housing*	*5* *56* *7*	*7* *82* *10*

^a^ Farmers could give several answers to these questions, therefore the sum of the percentages can exceed 100 %

^b^ The vaccinations were grouped based on whether the pathogen affected mainly reproductive performance (Porcine parvovirus (PPV), Porcine reproductive and respiratory syndrome virus (PRRSV) and *Erysipelothrix rhusiopathiae*), or respiratory (Swine influenza virus (SIV), Porcine circovirus type 2 (PCV-2), *Mycoplasma hyopneumoniae*, *Actinobacillus pleuropneumoniae*, *Pasteurella multocida* and *Bordetella bronchiseptica*, and *Glaeserella parasuis*) or intestinal health (Porcine rotavirus type A (PRVA), *Escherichia coli* and *Clostridium spp.*)

**Table 4:** Results of the continuous variables in the questionnaire

Variable	median	min	max
How long are you already working with the same origin farm (years)? (*n* = 18)	*5*	*1*	*12*
What is the frequency of purchasing breeding gilts (times per year)? (*n* = 39)	*6*	*1*	*13*
What is the age of the purchased breeding gilts (weeks)? (*n* = 39)	*24*	*9*	*37*
What is the minimum duration of the quarantine period (days)? (*n* = 37)	*42*	*14*	*140*
What is the stocking density of the breeding gilts kept in group housing (m²)? (*n* = 53)	*1.00*	*0.75*	*5.00*

For the entire questionnaire, except for the general information, there were five questions where numerical data were collected (Table 4). There were three variables for the part on purchasing policy: years of cooperation with the same origin farm, frequency of purchase and age of the purchased gilts; one variable for the part of the quarantine period: duration of quarantine; and one variable on the acclimation practices: stocking density of the breeding gilts in group housing.

The questions on the purchasing policy of breeding gilts are presented in Table 1. Table 2 shows the questions on the quarantine period of breeding gilts. If farmers indicated the presence of a quarantine unit, they had to clarify whether the quarantine unit was located externally, i.e. on a different site away from the farm, or internally, i.e. on the same site of the farm. They had to specify the type of stable as well; an isolated stable meant that the air volume and manure pit were completely separate. The all-in/all-out principle meant that new gilts could only enter the stable after the previous batch had moved to a new compartment. A separate hygiene lock was defined as a room where people could change their coverall and boots, and wash their hands, before entering the quarantine.

There were different questions for collecting information on acclimation practices (Table 3). The first one was related to the vaccination strategies that were used, for example, age at vaccination, where vaccinations were given (origin farm, purchasing farm), and the product used. Farmers who raised their own breeding gilts, could indicate as well which vaccinations were applied in the rearing unit. Pathogens against which vaccination is common and/or for which commercial vaccines are available, were considered. The different information on vaccination, including vaccination strategies, were classified in seven categories: no vaccination, one vaccination at the origin farm, one vaccination in the quarantine unit, more than one vaccination at the origin farm, more than one vaccination in the quarantine unit, combination of vaccinations at the origin farm and the quarantine unit, and vaccination without further details. Other acclimation practices, monitoring for pathogens, and questions on housing conditions of the breeding gilts, were included as well (Table 3).

### Distribution of the questionnaire

Belgian pig farmers were contacted and visited by veterinarians of the Unit of Porcine Health Management (*n* = 6), a veterinarian at Animal Health Care Flanders (*n* = 1), and by several pig practitioners (*n* = 4). The pig farmers were able to participate voluntarily, and the questionnaires were filled out during an on-farm interview linked to routine farm visits. Therefore, the selected farms are a convenience sample. The answers given by the farmer were considered to be accurate and were not verified. For most herd visits done by the Unit of Porcine Health Management and Animal Health Care Flanders, the herd veterinarian was present, which enhances the validity of the answers. Questionnaires were collected from 1 October 2019 until 31 March 2020.

### Analysis of the data

Statistical analysis was mainly performed using IBM® SPSS® Statistics for Windows Version 24 (IBM Corp., Armonk, N.Y., USA). Descriptive statistics were performed for both the continuous and the categorical variables of the questionnaire. For the continuous variables, the median, minimum and maximum values were determined. For the categorical variables, percentages were calculated. No categories were deleted; however, some categories were merged to simplify complex outcomes. Normality distribution was analysed graphically via histograms and Q-Q plots. A non-parametric Mann-Whitney U test was used to analyse potential differences between farms for the not normally distributed data, i.e. herd size and duration of the quarantine period. The Levene’s test was used for analysing equality of variances. A parametric independent samples t-test was used to analyse potential differences between farms for the normally distributed data, i.e. frequency of purchasing breeding gilts and number of different vaccinations in gilts. A Chi-Square test of independence was used to assess differences between categorical variables. *P* values below 0.05 were statistically significant.

The agreement between the observed measures and the described optimal procedures was analysed by means of a categorical variable decision tree. First, it was checked whether they applied all three purchasing principles, i.e. the same origin farm, high health status of origin farm, and requirements for the transport truck. Second, the quarantine building was checked, i.e. having a separate air volume, and having a separate hygiene lock for the quarantine unit. Third, the quarantine management was evaluated, i.e. using the all-in/all-out principle, and having a quarantine duration of minimum 28 days. If all previously mentioned procedures were applied, the farm was considered to comply with the optimal introduction procedures as described in the Background section.

## Results

### Participating pig herds

All contacted pig farmers (*n* = 68) completed the questionnaire. All farms were located in Flanders. The median (min. – max.) number of sows on the farms was 300 (85–2500). The 1-, 2-, 3-, 4- and 5-week batch management system were used on 14 % (10/68), 6 % (4/68), 31 % (21/68), 37 % (25/57), and 12 % (8/68) of the farms, respectively.

### Purchasing policy

The results on the purchasing policy of breeding gilts are shown in Tables 1 and 4. Breeding gilts were purchased on 57 % (39/68) of the farms, while the remaining 43 % (29/68) reared their own breeding gilts. Ninety-seven per cent (38/39) of the purchasing farms worked with the same origin farm each time they purchased breeding gilts. Only 47 % (18/38) of the latter indicated the duration of their cooperation with the same origin farm, which was in median (min. – max.) 5 (1–12) years.

Seventy-nine per cent (31/39) of the purchasing farmers claimed a health status of the origin farm that was higher than or equal to their own farm, whereas on 21 % (8/39) of the purchasing farms, the health status was lower or the farmers did not know the health status of the origin farm. There were specific requirements for transport on 64 % (25/39) of the purchasing farms. The most common requirement was that the truck was exclusively used for transport of breeding gilts (44 %, 11/25), followed by a cleaned and disinfected truck (28 %, 7/25), and transport each time from one origin farm to only one purchasing farm (24 %, 6/25). Other requirements for gilt transport for 16 % (4/25) and 12 % (3/25) of the farms were that it had to be done as first job of the day or specifically as first job on Monday, respectively. The median (min. – max.) frequency of purchasing breeding gilts was 6 (1–13) times per year. The median (min. – max.) age of the purchased gilts was 24 (9–37) weeks.

### Quarantine period

The results related to the quarantine period are shown in Tables 2 and 4. On 95 % (37/39) of the purchasing farms, a quarantine unit was present. The quarantine unit had an external location in 3 % (1/37) of the cases and an internal location in 97 % (36/37) of the cases. When an external quarantine was used, the gilts were subsequently housed in an internal quarantine unit afterwards, before they joined the sow population in the herd. The gilts were isolated in a separate stable on 62 % (23/37) of the farms, whereas on 35 % (13/37) of the farms, the gilts were housed in a separate compartment within a stable. Two of the latter farmers specified that there was a separate manure pit, and one farmer reported that there was a separate ventilation system in the quarantine compartment. Hence, 68 % (25/37) of the farms had a quarantine unit with a separated air volume; i.e. the farms with an external quarantine unit (*n* = 1), the farms with an isolated stable (*n* = 23), and the farms that indicated separate ventilation in the quarantine compartment (*n* = 1). For the farms with a quarantine unit (*n* = 37), the all-in/all-out principle was used for the quarantine unit on 86 % (32/37) of the farms, and a separate hygiene lock for the quarantine unit was present on 54 % (20/37) of the farms. The median (min. – max.) duration of the quarantine period was 42 (14–140) days.

### Acclimation practices

**Fig. 1:**
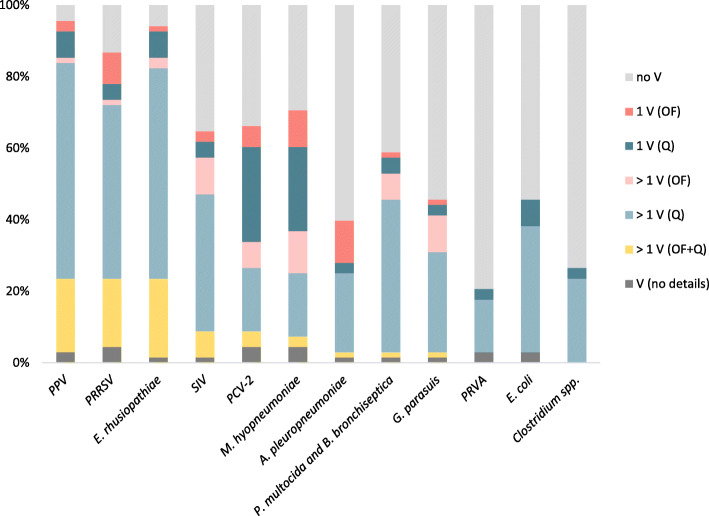
Vaccination strategies ^a^ (*n* = 7) used on all the farms (*n* = 68) for the different pathogens ^b^ (*n* = 12). ^a^ no V: no vaccination; 1 V (OF): one vaccination at the origin farm; 1 V (Q): one vaccination in the quarantine unit; > 1 V (OF): more than one vaccination at the origin farm; > 1 V (Q): more than one vaccination in the quarantine unit; > 1 V (OF + Q): combination of vaccinations at the origin farm and the quarantine unit; V (no details): vaccination without further details.  ^b^ The pathogens were grouped based on whether they affected mainly reproductive performance (Porcine parvovirus (PPV), Porcine reproductive and respiratory syndrome virus (PRRSV) and *Erysipelothrix rhusiopathiae*), or respiratory (Swine influenza virus (SIV), Porcine circovirus type 2 (PCV2), *Mycoplasma hyopneumoniae*, *Actinobacillus pleuropneumoniae*, *Pasteurella multocida* and *Bordetella bronchiseptica*, and *Glaeserella parasuis*) or intestinal health (Porcine rotavirus type A (PRVA), *Escherichia coli* and *Clostridium spp.*).

Table 3 shows the frequency of vaccination of breeding gilts against different pathogens. The vaccination strategies for each pathogen are shown in Fig. 1. Furthermore, two farmers vaccinated the gilts with an autogenous vaccine against *Streptococcus suis*, and on one of those farms *Staphylococcus hyicus* was included in the vaccine as well. The median (min. – max.) number of pathogens against which gilts were vaccinated was 7 (2–12).

Fifty-seven per cent (39/68) of the farms used one or more acclimation practices (Table 3). Giving faeces from suckling piglets to the gilts was used in 18 % (12/68) of the farms, followed by housing sows that will be culled in the same compartment as the breeding gilts (16 %, 11/68). Other acclimation practices included the provision of placenta tissue (9 %, 6/68), faeces from weaned piglets (3 %, 2/68) and faeces from piglets with diarrhoea (1 %, 1/68) to the breeding gilts. Several other acclimation practices were used as well (31 %, 21/68) such as giving faeces from sows, providing a burlap bag which hung first in the farrowing or nursery unit for contact with faeces or oral fluids, and giving leftovers from the feeding corridor to the breeding gilts. Eighteen per cent (12/68) of the farms used different combinations of acclimation methods. Seven per cent (5/68) of the farms indicated that they did own rearing of gilts, and that gilts were housed in a pen in the finishing or gestation unit.

Eighty-four per cent (57/68) of the farms did not monitor for any pathogen during the quarantine or acclimation period (Table 3). Two farms took faecal samples to monitor *Brachyspira hyodysenteriae* via PCR testing (3 %), while other farms monitored the presence of antibodies in serum against other pathogens using ELISA (10 %, 7/68), and two farms practiced the combination of both (3 %).

Table 3 shows the information on housing of breeding gilts. Breeding gilts were housed in different ways. On 7 % (5/68) of the farms, they were kept individually, while on 82 % (56/68) of the farms, the gilts were housed in groups. Ten per cent (7/68) of the farms used a combination of individual and group housing. The median (min. – max.) stocking density on the farms, where gilts were housed in groups, was 1.00 (0.75–5.00) m² per gilt. Farms were categorised in three different categories according to stocking density of breeding gilts: < 1 m² (8 %, 4/53), 1–1.5 m² (74 %, 39/53), and > 1.6 m² (19 %, 10/53).

### Differences between the herds

There was a statistically significant difference in herd size between farms that required hygienic measurements (median: 360 sows) and farms that did not require hygienic measurements of the transport vehicle (median: 220 sows) (*P* = 0.006). There was a statistically significant difference in duration of the quarantine period between farms that had a separate hygiene lock (median: 46 days) and farms that did not have a separate hygiene lock for the quarantine unit (median: 31 days) (*P* = 0.007). There was a significant increase in the frequency of purchasing breeding gilts by on average 4 times per year (95 % CI: 1 to 7, *P* = 0.023) for farms that did not use the all-in/all-out principle (10 ± 2 times per year) compared to farms that did use the all-in/all-out principle in the quarantine unit (6 ± 3 times per year). There was a significant decrease in number of pathogens against which gilts were vaccinated by on average 2 pathogens (95 %CI: -3 to -1, *P* = 0.004) for farms that reared their own gilts (6 ± 3 pathogens) compared to farms that purchased breeding gilts (8 ± 3 pathogens). No statistically significant associations were found between the purchasing of breeding gilts and the use of a batch management system (χ² = 0.000, *P* = 1.000), nor between the purchasing of breeding gilts and the use of acclimation practices (χ² = 0.655, *P* = 0.418).

### Optimal introduction procedures

For the purchasing farms, the compliance to the optimal introduction procedures was verified (Table 5). Fifty-four per cent (21/39) of the farms applied all three principles of the purchasing policy. Ninety-five per cent (37/39) of the farms housed their gilts in a quarantine unit. Thirty-eight per cent (14/37) of the farms with a quarantine unit had a proper quarantine building, i.e. a stable with a separate air volume (external or internal location) with a separate hygiene lock. Eighty-one per cent (30/37) of the farms managed the quarantine unit properly, i.e. using the all-in/all-out principle and having a quarantine duration of minimum 28 days. However, combined, only 10 % (4/39) of the farms complied with the optimal introduction procedures of all three categories, i.e. purchasing policy, quarantine building and quarantine management.

**Table 5:** Compliance of the farms to the optimal introduction procedures

Variable	n	%
Application of the correct procedures regarding the purchasing policy (*n* = 39)		
*Always working with the same origin farm* *High health status of the origin farm* *Hygienic requirements for the transport vehicle* *All of the above*	*38* *31* *25* *21*	*97* *79* *64* *54*
Application of the correct procedures regarding the quarantine building (*n* = 37)		
*Quarantine unit with separate air volume* *Hygiene lock for the quarantine unit* *All of the above*	*25* *20* *14*	*68* *54* *38*
Application of the correct procedures regarding the quarantine management (*n* = 37)		
*Application of the all-in/all-out principle* *Quarantine period duration of minimum 28 days* *All of the above*	*32* *34* *30*	*86* *92* *81*

## Discussion

This study investigated the introduction procedures of breeding gilts in Belgian pig farms, and more specifically which purchasing, quarantine and acclimation practices pig farmers use. Furthermore, current field practices were contrasted with the optimal introduction procedures.

A lot of attention was paid to the wording of the questions and structure of the questionnaire. The questionnaire was pre-tested by colleagues and experts in the field. Despite the fact that the questionnaires were filled in during an on-farm interview, it was for some questions unclear how they were interpreted precisely by the veterinarian and/or the farmer. Regarding the vaccination strategies for instance, it was unclear whether the answers related to the rearing phase and quarantine period only, or whether vaccinations applied during the first gestation of the gilts were included as well. The same was true for the housing of the gilts. Nevertheless, the answers were assumed to be applicable to the rearing and quarantine phase, and they were not excluded from the analysis. The absence of the answering option ‘sometimes’ for some questions is another limitation of the questionnaire design. By only providing the options ‘yes’ and ‘no’, it is assumed that farmers always work according to the same principles, which might not necessarily or always be the case.

Pig production in Belgium is mostly located in Flanders, the northern Dutch-speaking part of the country. In 2020, there were approximately 6.1 million pigs in Belgium: 1.6 million piglets (< 20 kg), 4 million fattening pigs (> 20 kg), and 400.000 sows. Ninety-seven per cent of the sows were located in Flanders, and only 3 % in Wallonia, the southern French-speaking part of the country [11]. All questionnaires were collected in Flanders. For 2019, the number of herds are known as well. The sows in Belgium were housed on 1.678 different farms, of which 90 % (*n* = 1518) located in Flanders, and 10 % (*n* = 160) in Wallonia [12]. In Flanders, 60 % (911/1518) of the sow herds had more than 150 pigs, while in Wallonia, only 16 % (25/160) of the sow herds had more than 150 pigs [13]. In terms of herd size, the sow herds in the present study were representative for other sow herds in Belgium [3, 14, 15]. Also the batch management systems of the farms in the present study were in line with other studies, showing that the 3- and 4-week batch management system are most commonly used in Belgium [14–16].

Garza-Moreno et al. [7] found that in Europe replacement gilts were purchased from another farm in 45 % of the cases, whereas own rearing of gilts occurred on 32 % of the farms. On the remaining 23 % of the farms, there was a combination of purchasing and own rearing of gilts. Chantziaras et al. [17] found similar percentages: 56 % of the farms purchased breeding gilts and 44 % reared own gilts. Caekebeke et al. [15] found that more than half of the Belgian farms did not purchase any animals. In our study, we found that 57 % (39/68) of the farms purchased breeding gilts and 43 % (29/68) bred their own gilts, and no combination of these methods was used. Purchasing breeding gilts is a risk factor for introduction of pathogens into the farm [2], for example Porcine reproductive and respiratory syndrome virus (PRRSV). [18] Three per cent (1/39) of the farms indicated that the breeding gilts originated from multiple origin farms, which is generally considered as a clear risk. Purchasing breeding gilts from multiple origin farms increases the risk of reinfection of specific-pathogen-free (SPF) farms with *M. hyopneumoniae*, and can result in a large number of slaughter pigs seropositive for *A. pleuropneumoniae* serovar 2 [19, 20].

Transport vehicles of livestock are found to be an important source of contamination for many pathogens, such as classical swine fever [21], *M. hyopneumoniae* [22], * A. pleuropneumoniae* [22], and * B. hyodysenteriae* [23]. Therefore, transport vehicles should be cleaned and disinfected according to a strict protocol before they are allowed to enter the premises [8]. However, only 28 % (7/25) of the farms indicated cleaning and disinfection as a requirement for the transport truck. In addition, 28 % (7/25) of the farmers only allowed transport vehicles on their farm in the morning when no other farms had been visited, or on Monday morning when no other farms had been visited in the weekend. These requirements consider that in these cases, transport vehicles are clean and disinfected, and were empty for at least 12 hours.

On 5 % (2/39) of the farms, it was indicated that the purchased animals were not isolated in a quarantine unit. One of those farmers specified that the gilts were housed immediately in a compartment where other pigs of the farm were present as well, and this farmer was aware of the risk associated with this procedure. The reason they did not use a quarantine was not asked. Sixty-eight per cent (25/37) of the farms had a quarantine unit where the air volume was separated from other pigs on the farm, namely the farms with an external quarantine unit, the farms with an isolated stable, and the farms that indicated that there was a separate ventilation system in the quarantine compartment. Isolation of purchased breeding gilts can reduce the risk of pathogen introduction in the herd [18]. In the North American swine industry it is common to house breeding gilts in a specialised gilt development unit (GDU). GDUs are used to raise gilts and to gradually adapt them to the health status of the sow herd [24].

Pathogens can be transmitted indirectly through contaminated hands, clothing and boots [3] Therefore, it is important to have a separate hygiene lock for the quarantine unit, with water supply for hand hygiene, and specific clothing and boots for the quarantine unit. Only 54 % (20/37) of the farms had a separate hygiene lock for the quarantine, and it is not known when hygiene measures were taken if they were performed well according to a strict protocol. Pathogen transmission between gilts in the quarantine unit and other animals on the farm can occur when no measures are taken in between entering the quarantine unit and the compartments of the other animals [2]. Risk of pathogen transmission depends on whether people visit the quarantine unit as the last task of the working day, and if they start the next day with clean clothing and cleaned and disinfected boots. This is especially important in the initial phase of the quarantine period, when the main goal is avoiding possible introduction of pathogens by newly purchased animals into the farm [25]. The frequency and duration of visits to the quarantine unit by the farmer and/or employees could have as well an influence on this. Moreover, this information is important to estimate whether monitoring for clinical signs in the breeding gilts was performed properly. Nevertheless, information on frequency, duration, and time point of visits to the quarantine unit was not included in the questionnaire.

Pritchard et al. [2] suggested that the quarantine period should last at least three to four weeks. According to Neumann and Hall [6] the duration of the quarantine period typically varies between 30 and 60 days. Both studies agree that the duration of the quarantine period depends on the specific pathogens of concern. In Belgian pig production, there is limited legislation on the application and duration of a quarantine period. Article 7 of the Belgian Royal Decree of 18 June 2014, regarding measures to prevent notifiable swine diseases, states that farms are the first four weeks after purchasing of new animals only allowed to transport finishing pigs to the slaughterhouse. However, if a quarantine period of four weeks is applied for the newly purchased animals, the farms are allowed to send piglets to other farms, or to transport sows that will be culled to the slaughterhouse [26]. Since the threat of African Swine Fever (ASF) in Belgium, extra legal requirements were in place, which are listed in the Ministerial Decree of 26 September 2018 regarding urgent measures to control ASF. Chapter 2 of this decree includes biosecurity measures for the entire country, for example, all pigs that enter a herd must be housed separately for four weeks (Article 15), and group treatment of clinically sick animals is only allowed after a negative ASF-diagnosis is confirmed by laboratory analysis (Article 16) [27]. The median duration of the quarantine period in this study was 42 days, which proves that 50 % of the farms had a quarantine period longer than or equal to six weeks, which should be long enough to monitor clinical signs of several diseases, and to perform laboratory testing. However, previous studies have shown that monitoring for diseases during the quarantine period is not very common. In the study of Garza-Moreno et al. [7], 28 % of the farms performed diagnostics for *M. hyopneumoniae* in the purchased breeding gilts. Lambert et al. [28] found that 11 % of the farms evaluated the PRRSV-status of the gilts at the end of the quarantine period. In our study, only 16 % (11/68) of the farmers monitored breeding gilts for presence of *B. hyodysenteriae* (via PCR) and/or other pathogens (via ELISA). Furthermore, interpretation of serological data requires knowledge on vaccination of the pigs (vaccination yes/no, product used, scheme), since most serological tests make no distinction between antibodies originating from infection or vaccination [29]. To this end, it is important to know if the purchased animals already received vaccinations at the origin farm. For all pathogens, vaccination was done at the origin farm, except for the pathogens related to intestinal health, i.e. porcine rotavirus type A, *Escherichia coli*, and *Clostridium spp*. A possible explanation could be that breeding gilts only receive those vaccinations at the end of first gestation, in order to provide their offspring with lactogenic immunity. Other techniques can be used as well to detect the pathogen, or antigens, or genetic material in blood, tonsil samples, nasal samples, laryngeal and tracheal swabs, fluid of bronchoalveolar lavage, and faeces [29]. Assessing the health status of the breeding gilts by clinical evaluation and laboratory testing, provides valuable information to the farmers, as it could help them to prevent pathogen introduction and/or maintain a free-status for specific pathogens, for example *B. hyodysenteriae*. In total, 79 % (31/39) of the farmers was aware of the health status of the origin farm, hence they knew for which pathogens these farms were free. Purchasing gilts from an SPF farm could be useful as well to keep the farms free of specific diseases. This can be considered as primary disease prevention, since the main goal is to avoid introduction of specific pathogens in a farm [30]. The number of SPF farms in Belgium is not known, in contrast to Denmark where 2,300 sow herds (80 % of the Danish sows) have an SPF health status (TS Hansen, personal communication).

Vaccination of purchased breeding gilts was practiced in all farms and was therefore the most commonly used acclimation practice. A variety of vaccination strategies were used, and on all farms, breeding gilts were vaccinated against at least two pathogens. This is in accordance to Garza-Moreno et al. [7], who found that vaccination was the most important gilt acclimation practice for *M. hyopneumoniae* in Europe. Most vaccines do not give a full protection, do not prevent infection and cannot eliminate pathogens from a herd. Nevertheless, vaccination is very important because it reduces the risk of pathogen transmission, clinical signs, lesions, and performance losses due to disease [29]. Subsequently, vaccination may be cost-efficient, even in subclinically infected herds [31]. Pieters and Fano [32] suggested a method of strategic exposure of gilts to *M. hyopneumoniae* by vaccination at a young age, aiming to let them undergo the infectious process, and recover and gain immunity. In this way, these animals do not shed *M. hyopneumoniae* anymore when they are introduced in the sow herd. For some pathogens (e.g. *B. hyodysenteriae*, * S. suis*) no commercial vaccines are available, and sometimes autogenous vaccines are used. However, little is known on the efficacy and safety of these vaccines [33]. In this study, two farmers used autogenous vaccines.

Bringing faeces from piglets in the rearing or quarantine unit was sometimes used as an acclimation practice. Interpreting Article 36 and 39 of the Regulation (EC) No 1069/2009 regarding animal by-products, faeces could be used as a derived product for disease prevention by controlled exposure if advised by the veterinarian. However, faeces cannot be used as a source of nutrition for animals, and pig feed and drinking water should be kept clean and safe at all time [34]. It is better not to use alternative acclimation practices, such as contact with faeces or placenta tissue, since it is not known which pathogens are in, and thus it is unclear which pathogens are spread. Therefore, it is in contrast with internal biosecurity principles, aiming to prevent or limit pathogen transmission within the herd [25].

On 82 % (56/68) of the farms, the breeding gilts were housed in groups. On 8 % (4/53) of those farms, the stocking density of the breeding gilts was less than 1 m². This is not in accordance with the minimal legal requirements, which state that the surface area per pig, weighing more than 110 kg, should be 1 m², and preferably even higher [35]. Moreover, higher stocking density can lead to a higher level of disease, and can predispose to leg weaknesses and claw disorders [36, 37].

If the separate categories of the introduction procedures are considered, namely purchasing policy, quarantine building and quarantine management, 54 % (21/39), 38 % (14/37) and 81 % (30/37) of the farms respectively, complied with the optimal procedures. However, only 10 % (4/39) of the farms complied with all optimal introduction procedures. This indicated that there is a lot of room for improvement, and efforts should be made to raise the farmers’ awareness. Herd veterinarians could play a key role in improving biosecurity related to the introduction procedures of breeding gilts in pig herds. This could be facilitated by the use of a checklist or a step-wise protocol for the purchasing of breeding gilts.

## Conclusions

Fifty-seven per cent (39/68) of the farms purchased breeding gilts, and there was a lot of variation in the frequency of purchase and the age at which gilts are purchased. On 95 % (37/39) of those farms, a quarantine unit was used, where on most farms the quarantine was located on the farm itself. In general, the gilts were kept in quarantine for six weeks. Vaccination was the most commonly applied acclimation practice, although in some farms exposure of gilts to farm-specific micro-organisms was done by providing faeces of suckling piglets and bringing the gilts in contact with sows before culling. Only 10 % (4/39) of the farms applied the optimal introduction procedures of breeding animals, so there is a lot of room for improvement, and farmers’ awareness on this topic should be raised.

## Data Availability

The datasets used and/or analysed during the current study are available from the corresponding author on reasonable request.
